# Preoperative coronal tibiofemoral subluxation does not affect 2-year outcomes after mobile-bearing medial unicompartmental knee arthroplasty for anteromedial osteoarthritis

**DOI:** 10.1007/s00402-026-06530-x

**Published:** 2026-10-29

**Authors:** Tomofumi Kinoshita, Kristine Ifigenia Bunyoz, Kristian R. L. Mortensen, Christian Bredgaard Jensen, Andrew Price, Kirill Gromov, Anders Troelsen

**Affiliations:** 1 Department of Orthopaedic Surgery, Copenhagen University Hospital Hvidovre, Copenhagen, Denmark; 2 Department of Orthopaedic Surgery, Ehime University Graduate School of Medicine, Toon, Japan; 3 Nuffield Department of Orthopaedics, Rheumatology, and Musculoskeletal Sciences, University of Oxford, Oxford, United Kingdom; 4 Department of Clinical Medicine, University of Copenhagen, Copenhagen, Denmark

**Keywords:** Unicompartmental knee arthroplasty, Mobile-bearing, Coronal tibiofemoral subluxation, Oxford knee score

## Abstract

**Introduction:**

Coronal tibiofemoral subluxation (CTFS) is frequently observed in patients with medial knee osteoarthritis; however, its clinical relevance and the acceptable degree of CTFS when considering unicompartmental knee arthroplasty (UKA) remain unclear. This study aimed to determine whether preoperative CTFS influences short-term postoperative clinical outcomes in patients with anteromedial osteoarthritis (AMOA) undergoing medial UKA.

**Methods:**

In this retrospective cohort study, 457 consecutive knees with AMOA who underwent mobile-bearing UKA performed at a single center between August 2016 and May 2023 were analyzed. CTFS, defined as medial translation of the femur relative to the tibia, was measured on preoperative weight-bearing anteroposterior radiographs and expressed as the percentage of tibial plateau width. Patients were categorized into mild (0–5%), moderate (≥ 5% to < 10%), and severe (≥ 10%) CTFS groups. Clinical outcomes were evaluated using the Oxford Knee Score (OKS) preoperatively and at 3 months, 1 and 2 years postoperatively. Pairwise comparisons of changes in OKS were performed using a general linear model with Bonferroni-adjusted post hoc tests, and adjusted mean differences were calculated controlling for age, sex, body mass index, and preoperative OKS.

**Results:**

At 2 years, mean OKS improvements were 16.6 ± 8.9, 14.8 ± 8.3, and 16.0 ± 9.3 in the mild, moderate, and severe CTFS groups, respectively. Adjusted mean differences between groups were small, with confidence intervals including zero. The severe CTFS group demonstrated greater joint line convergence angle than the mild and moderate groups (both *p* < 0.001). Mean bearing thickness did not differ among groups (*p* = 0.428).

**Conclusions:**

Preoperative CTFS was not associated with worse outcomes in mobile-bearing UKA for AMOA with correctable deformity. Within the setting of correctable deformity and appropriate patient selection, preoperative CTFS was not associated with inferior 2-year outcomes. Longer-term survivorship and radiographic progression analyses are warranted.

**Supplementary Information:**

The online version contains supplementary material available at https://doi.org/10.1007/s00402-026-06530-x.

## Introduction

Coronal tibiofemoral subluxation (CTFS) refers to medial translation of the femur relative to the tibia on the coronal plane and is frequently observed in patients with medial compartment knee osteoarthritis. It has been reported to be associated with the coexistence of marked varus deformity and anterior cruciate ligament insufficiency in advanced knee osteoarthritis [1] and has emerged as a radiographic parameter of increasing clinical interest [2]. However, its clinical relevance in patients with anteromedial osteoarthritis (AMOA) receiving mobile bearing unicompartmental knee arthroplasty (UKA) remains unclear.

Previous studies have suggested potential associations between this subluxation and preoperative functional impairment [3]. In total knee arthroplasty (TKA), CTFS has also been reported to contribute to intraoperative ligament balance [4]. In contrast, regarding UKA outcomes in patients with AMOA, previous studies have reported no adverse influence, whereas others suggest that uncorrectable subluxation may negatively affect postoperative results [5, 6]. However, these studies are limited by small sample sizes, and robust evidence in larger, stratified cohorts is lacking. This issue may be particularly relevant in mobile-bearing UKA because the mobile-bearing design is less constrained than fixed-bearing implants and may therefore be more sensitive to preoperative joint morphology and ligament balance, including tibiofemoral subluxation. Clarifying the role of preoperative CTFS may improve surgical decision-making and patient selection for UKA, as the acceptable preoperative extent of this subluxation has not been clearly defined.

Patients with lateral tibiofemoral osteoarthritis who undergo surgery have been reported to show greater progression of both malalignment and tibiofemoral subluxation compared with those managed conservatively [7]. However, no large-scale study has evaluated alignment or joint morphology specifically in patients undergoing UKA for AMOA. Consequently, the relationship between CTFS and alignment in this population remains poorly understood. CTFS may be related to coronal plane alignment and joint balancing, both of which are important factors in intraoperative decision-making. Excessive CTFS may alter ligament tension and load distribution across the tibiofemoral joint, potentially affecting implant stability, postoperative biomechanics, and long-term joint integrity. In TKA, subluxation can lead to unpredictable gap assessment, thereby increasing the relative reliability of bearing thickness [4]. No previous study has investigated the association between the degree of CTFS and the bearing thickness selected during surgery in UKA.

Given these uncertainties, we classified patients with AMOA according to CTFS severity and evaluated its impact on postoperative outcomes following mobile-bearing UKA. We also compared preoperative radiographic characteristics and bearing thickness, and hypothesized that preoperative CTFS would not adversely affect outcomes.

## Methods

### Patient selection

**Fig. 1:**
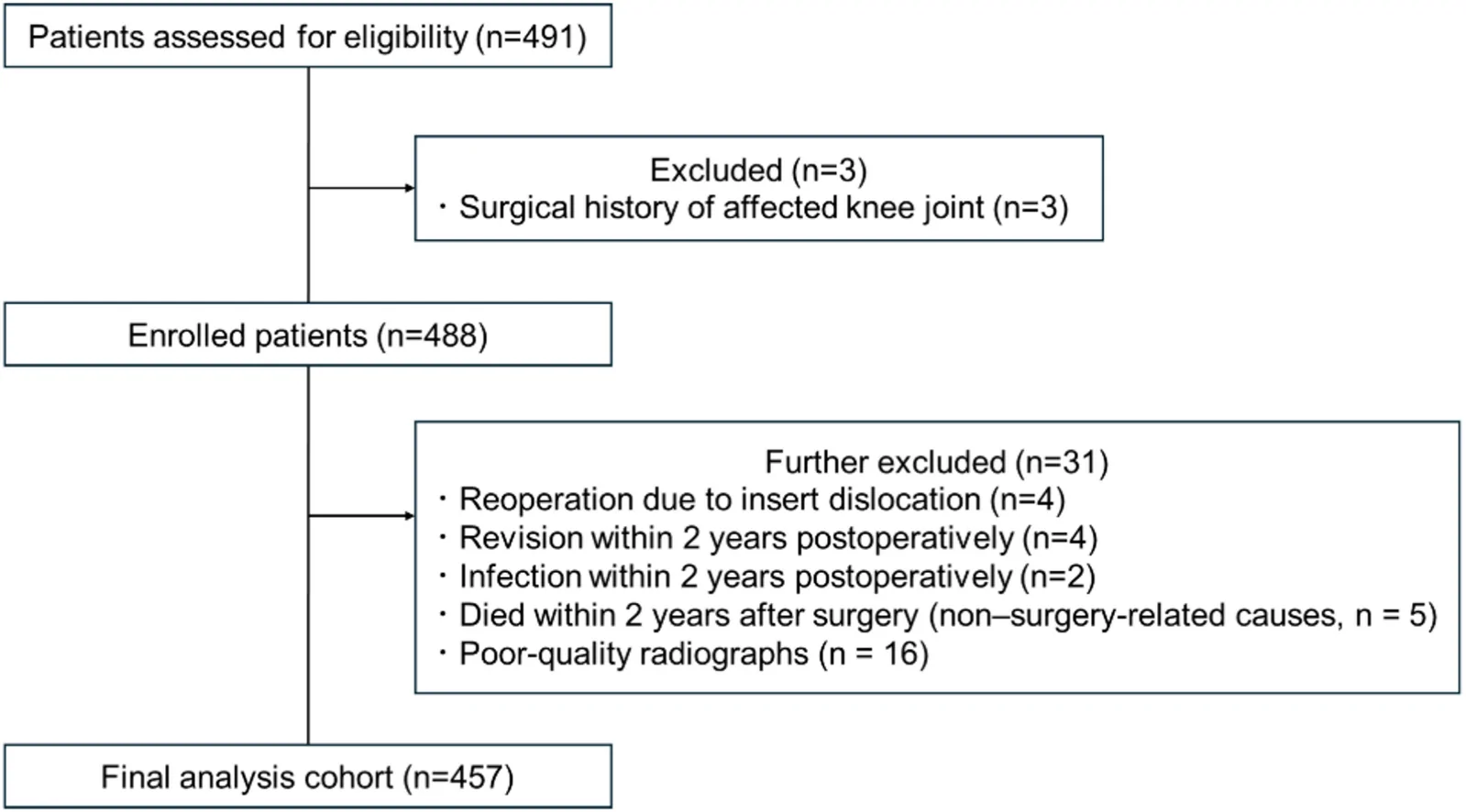
Flow diagram of patient inclusion and exclusion

Patients with AMOA who underwent primary mobile-bearing UKA performed by two surgeons (KG and AT) between August 2016 and June 2023 at a single center and who provided informed consent (*n* = 491) were retrospectively identified. The diagnosis of AMOA was based on previously described indications for medial UKA [8], including clinical symptoms and radiographic findings demonstrating medial compartment osteoarthritis with preserved lateral joint space, absence of posterior femoral subluxation on lateral radiographs, radiographic evidence of anteromedial tibial cartilage wear, functionally intact ligaments, and absence of inflammatory arthritis. The indication of surgery being AMOA implied that the intra-articular varus deformity was correctable. Correctable deformity was assessed preoperatively through clinical examination to confirm passive correction toward neutral alignment. Ligament integrity was determined based on manual assessment of joint stability, and no additional imaging such as MRI was routinely performed. All eligible patients were consecutively included during the study period to minimize selection bias, and no additional selection criteria were applied. Surgical indications and operative techniques, including ligament balancing, remained consistent throughout the study period. The exclusion criteria were a history of ipsilateral knee surgery (*n* = 3), reoperation within 2 years of follow-up (bearing dislocation, *n* = 4; infection, *n* = 2; revision surgery, *n* = 4: supplementary Table 1), death within 2 years of surgery due to non–surgery-related causes (*n* = 5), and suboptimal radiographic imaging (*n* = 16). In total, 457 knees were included (Fig. 1). Preoperative patient characteristics are shown in Table 1. All procedures were performed using the cementless Oxford Phase 3 mobile-bearing UKA (Zimmer Biomet, Oxford, UK) with microplasty instrumentation through a minimally invasive medial parapatellar approach without patellar eversion.

**Table 1:** Preoperative patient characteristics

	Total (*n* = 457)	Mild CTFS group (*n* = 134)	Moderate CTFS group (*n* = 265)	Severe CTFS group (*n* = 58)	*p*-value
Sex (female/male)	268/189	73/61	159/106	36/22	0.487
Age (years)	66.2 ± 9.6	66.3 ± 9.5	66.0 ± 9.9	66.4 ± 8.5	0.901
Height (cm)	171.5 ± 10.4	173.1 ± 9.9	171.2 ± 10.6	168.5 ± 10.4	0.027*
Weight (kg)	90.5 ± 19.4	92.9 ± 19.4	90.7 ± 19.1	83.9 ± 19.3	0.008*
BMI	31.0 ± 6.1	92.9 ± 19.4	30.7 ± 5.9	29.5 ± 6.3	0.174

Data are presented as mean ± standard deviation (SD). Categorical variables were compared using the chi-square test. Continuous variables were compared using Kruskal–Wallis test

*CTFS* coronal tibiofemoral subluxation, *BMI* body mass index

*p < 0.05

### Radiographic evaluation and outcome measures

**Fig. 2:**
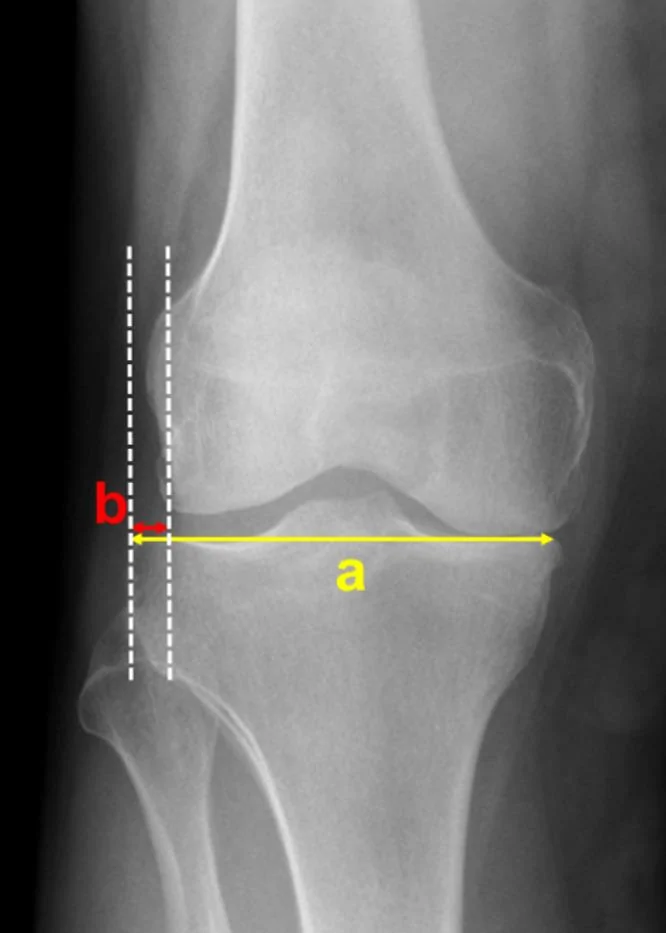
Measurement of coronal tibiofemoral subluxation (CTFS) on anteroposterior radiographs. CTFS was calculated as b/a × 100%, where a represents the tibial plateau width and b the lateral femoral displacement, excluding osteophytes

**Fig. 3:**
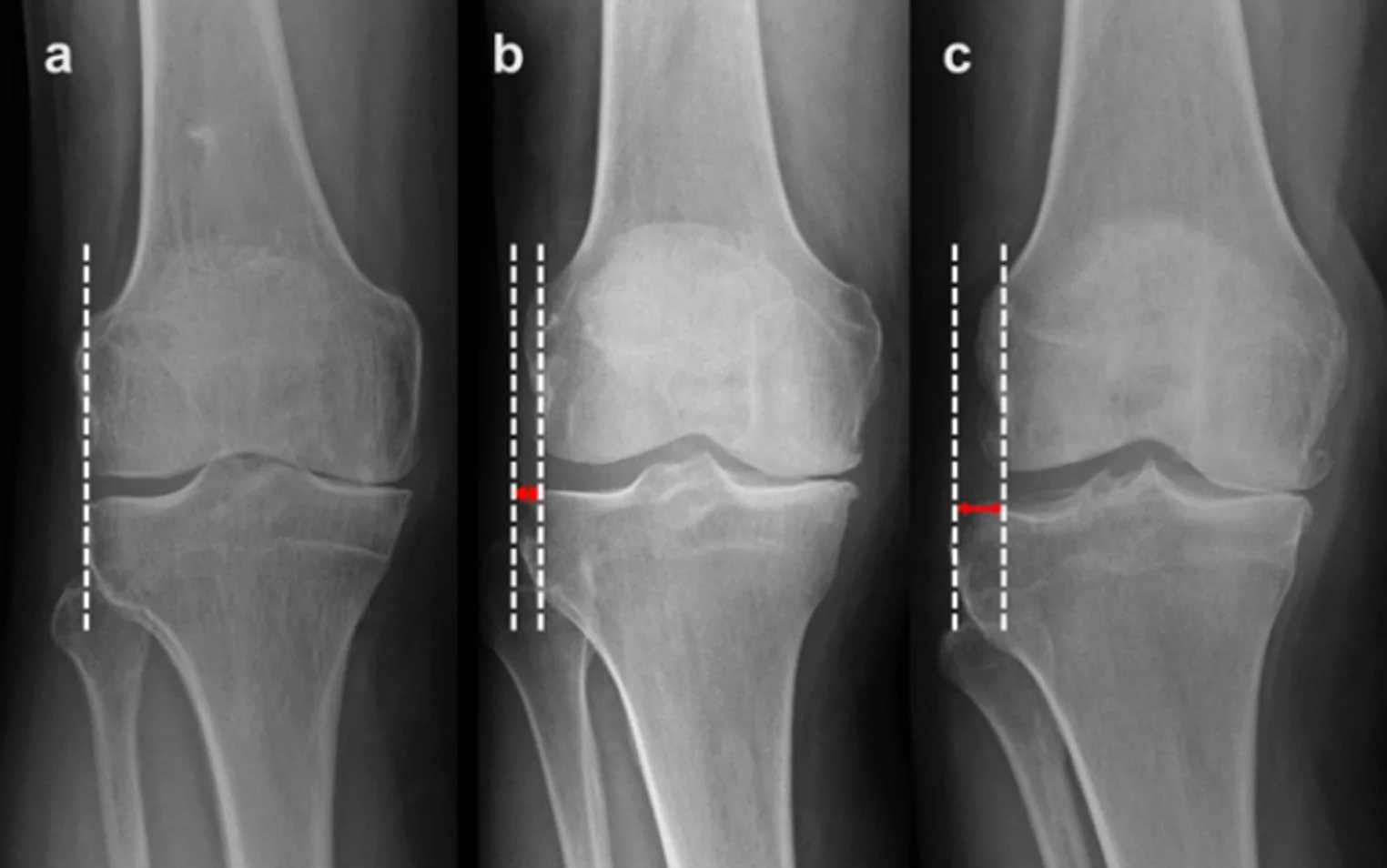
Representative radiographs illustrating coronal tibiofemoral subluxation (CTFS) categories. (**a**) Mild CTFS group (0–5%): No or minimal medial displacement of the lateral femoral condyle. (**b**) Moderate CTFS group (≥ 5% to < 10%): Evident medial displacement within the moderate range. (**c**) Severe CTFS group (≥ 10%): Marked medial displacement of the lateral femoral condyle relative to the tibial plateau

Short anteroposterior and lateral knee radiographs were obtained preoperatively. All radiographs were obtained using a standardized protocol with patients in the standing weight-bearing position and the patella facing forward. Short knee radiographs were used because they are routinely obtained for preoperative assessment and surgical planning for UKA at our institution. The femorotibial angle (FTA), the lateral distal femoral angle (LDFA), the medial proximal tibial angle (MPTA), joint line convergence angle (JLCA) and posterior tibial slope (PTS) were assessed. For consistency, valgus alignment was defined as negative and varus alignment as positive on the anteroposterior radiographs, whereas the posterior slope was defined as positive and the anterior slope as negative on the lateral radiographs. CTFS was measured in accordance with previous studies by quantifying the medial displacement of the lateral femoral condyle relative to the total width of the tibial plateau [1] (Fig. 2). Osteophytes were excluded, and the distance was measured from the true cortical margins of the lateral femoral condyle and the tibial plateau. CTFS was quantified as the subluxation percentage, calculated by (medial displacement/tibial plateau width) × 100. Knees were then categorized into mild (0–5%), moderate (≥ 5% to < 10%), and severe (≥ 10%) CTFS groups (Fig. 3). Because no universally accepted CTFS thresholds exist for UKA, the cut-offs were predefined before analysis to create clinically interpretable severity groups while maintaining adequate sample sizes in each category. In addition, both absolute OKS values at each time point (preoperatively, 3 months, 1 year, and 2 years) and changes from baseline were compared across the groups. OKS data were prospectively collected as part of routine clinical follow-up using standardized questionnaires and retrospectively analyzed for the purposes of this study. The OKS was collected via email or postal mail, with follow-up rates of 84.6%, 90.5%, 87.3%, and 84.0% at preoperative, 3-, 12-, and 24-month time points, respectively. The minimal clinically important difference (MCID) for the OKS has been reported to be approximately 5 points [9, 10]. Radiographic measurements were performed without blinding to group allocation but were blinded to clinical outcomes.

### Statistical analysis

To evaluate intraobserver and interobserver reproducibility of subluxation measurements, assessments were performed twice by one surgeon at intervals of more than 1 month in 100 cases and once by a second examiner on ten randomly selected knees from the study cohort [11]. For the primary analysis, radiographic measurements were performed once by a single observer. The intraclass correlation coefficient (ICC; two-way mixed-effects model, consistency, single measures) for intraobserver reproducibility was 0.864 (95% CI: 0.768–0.916, *p* < 0.001). The corresponding interobserver ICC (two-way random-effects model, absolute agreement, single measures) between the two examiners was 0.836 (95% CI: 0.467–0.957, *p* < 0.001). Data distribution was assessed for normality using the Shapiro–Wilk test, and homogeneity of variance was evaluated prior to analysis. Statistical tests were selected accordingly based on these assumptions. Missing PROM data were not imputed; all analyses were conducted using available-case analysis at each time point. A sensitivity analysis including only knees with complete 2-year OKS follow-up data was additionally performed. Pairwise comparisons of changes in OKS among groups were performed using a general linear model with Bonferroni-adjusted post-hoc tests. Adjusted mean differences (95% confidence intervals) were calculated after controlling for age, sex, body mass index, and preoperative score. Covariates were selected a priori based on their established clinical relevance and potential association with postoperative outcomes. Correlations between CTFS severity and radiographic parameters, as well as bearing thickness, were assessed using Spearman’s rank correlation coefficient. Bearing thickness was compared among the mild, moderate, and severe CTFS groups using the Kruskal–Wallis test. All statistical analyses were two-tailed, and a p-value < 0.05 was considered statistically significant. Statistical analyses were performed using SPSS Statistics version 28.0 (IBM Corp., Armonk, NY, USA).

### Ethical considerations

This retrospective cohort study was conducted in accordance with the Declaration of Helsinki. Data collection was approved by the Knowledge Centre on Data Protection Compliance, Capital Region of Denmark (P-2022-290), and patients provided informed consent for researchers to access their X-rays and electronic patient records.

## Results

### Clinical outcomes

**Fig. 4:**
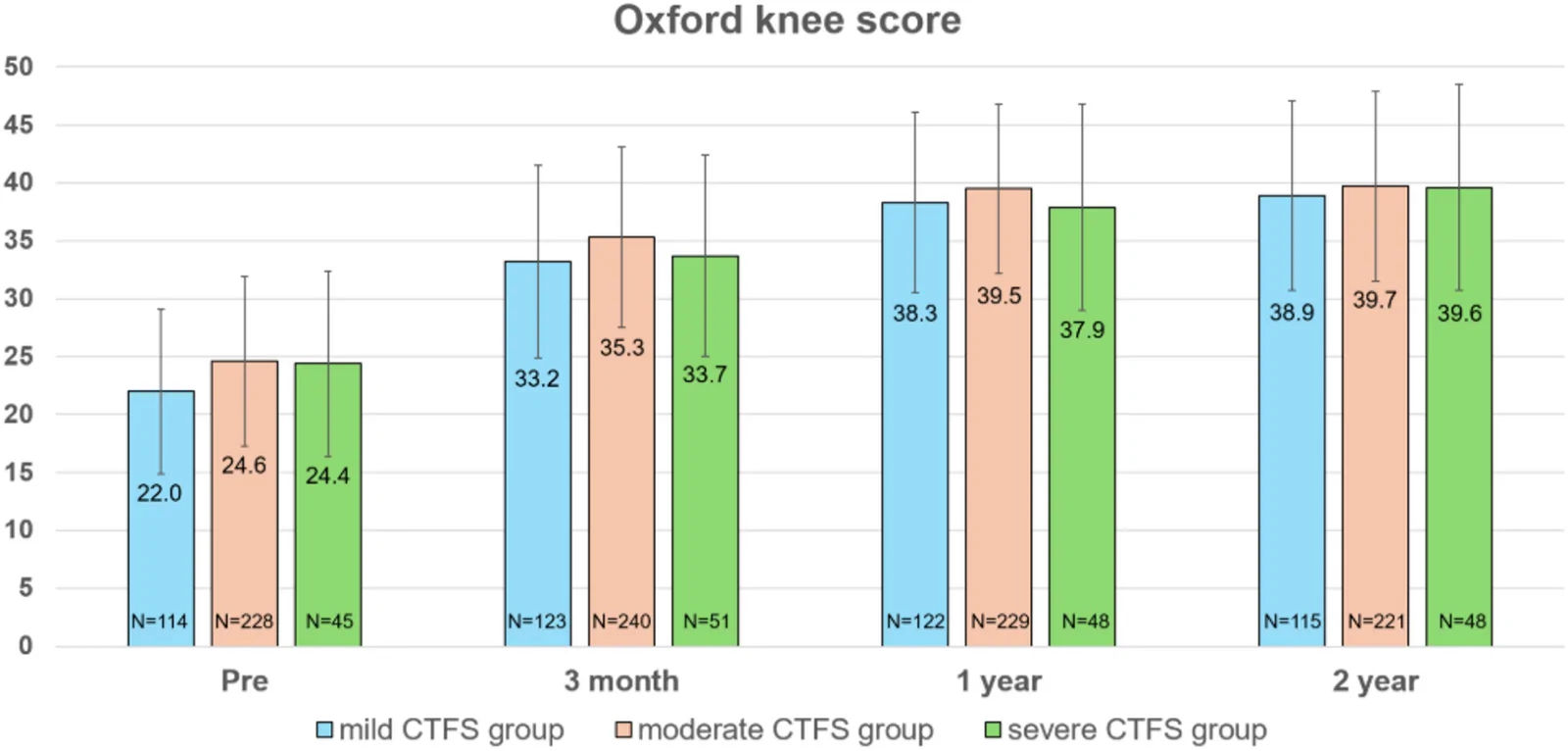
Comparison of mean Oxford Knee Score among each CTFS group at each time point. No significant differences were observed among the CTFS groups at any time point

**Fig. 5:**
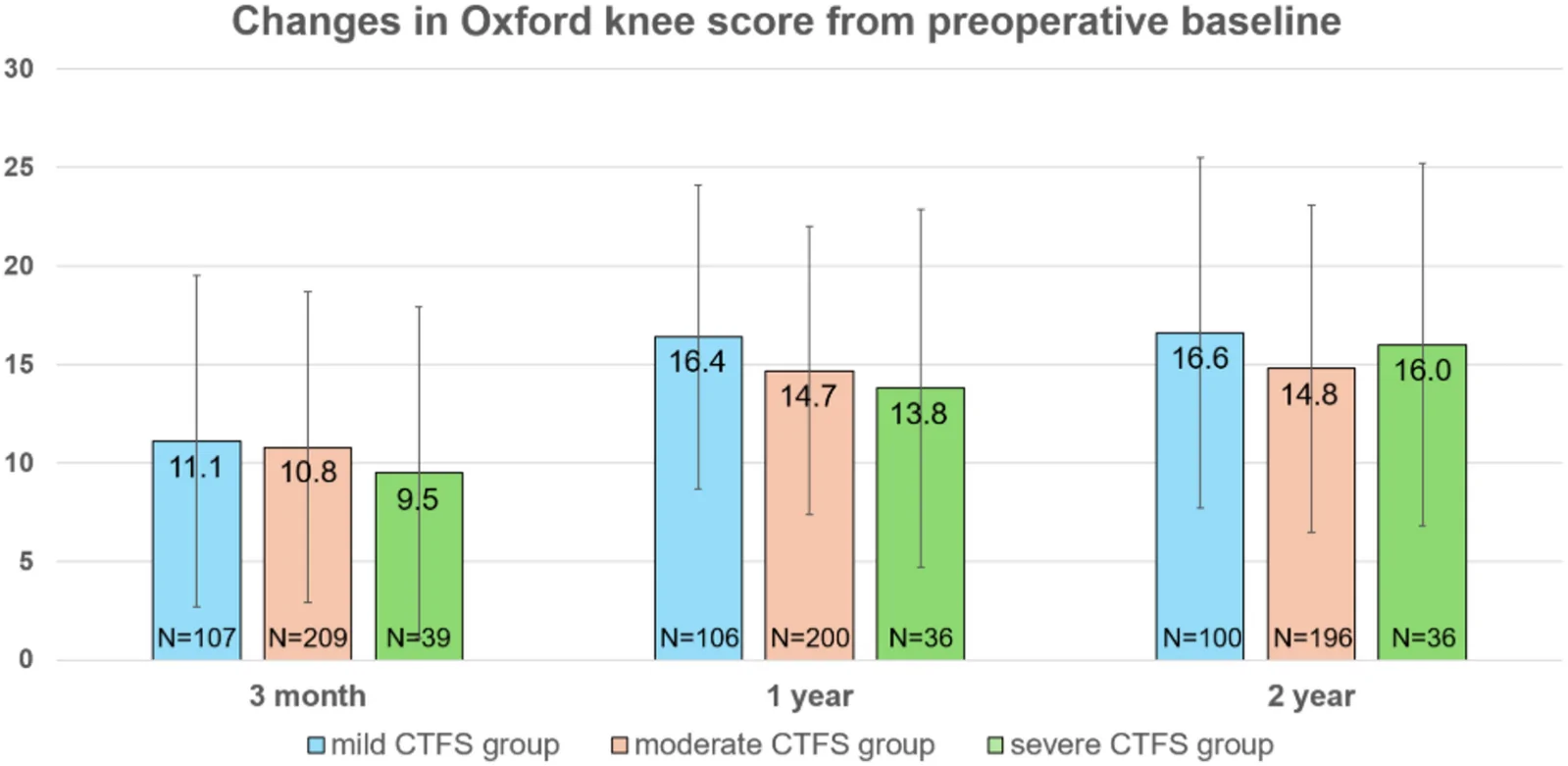
Comparison of mean changes in Oxford Knee Score from preoperative score among each CTFS group. No significant differences were observed among the CTFS groups at any time point

Absolute OKS values at each time point are presented in Fig. 4. At 2 years, mean OKS improvements were 16.6 ± 8.9, 14.8 ± 8.3, and 16.0 ± 9.3 in the mild, moderate, and severe CTFS groups, respectively. Improvements from baseline were comparable across groups, with small adjusted mean differences and confidence intervals including zero (Fig. 5; Tables 2 and 3). Mean OKS improvements in all CTFS groups exceeded the reported MCID threshold, whereas observed between-group differences remained below the reported MCID threshold for OKS. Detailed follow-up rates are provided in Supplementary Table 2. Sensitivity analysis including only knees with complete 2-year OKS follow-up data demonstrated findings consistent with the primary analysis, with no significant differences observed among the CTFS groups.

**Table 2:** Changes in Oxford Knee Score in the mild, moderate, and severe CTFS groups after medial UKA

	Mild CTFS group (number)	Moderate CTFS group (number)	Severe CTFS group (number)
Changes in OKS at 3 months after UKA	11.1 ± 8.4 (107)	10.8 ± 7.9 (209)	9.5 ± 8.4 (39)
Changes in OKS at 1 year after UKA	16.4 ± 7.6 (106)	14.7 ± 8.2 (200)	13.8 ± 9.1 (36)
Changes in OKS at 2 years after UKA	16.6 ± 8.9 (100)	14.8 ± 8.3 (196)	16.0 ± 9.3 (36)

Data are presented as mean ± standard deviation (SD)

*CTFS* coronal tibiofemoral subluxation, *OKS* Oxford Knee Score, *UKA* unicompartmental knee arthroplasty

**Table 3:** Pairwise comparisons of changes in OKS among CTFS severity groups after medial UKA

	Mean difference (95% CI)	*p*-value (Bonferroni adj.)	Adjusted (95% Cl)	*p*-value (Bonferroni adj.)
OKS				
**3month**				
Mild vs. moderate	0.238 (− 2.085 to 2.561)	> 0.99	−1.013 (− 3.092 to 1.066)	0.726
Moderate vs. severe	1.296 (− 2.112 to 4.704)	> 0.99	1.142 (− 1.928 to 4.213)	> 0.99
Mild vs. severe	1.534 (− 2.121 to 5.189)	0.940	0.129 (− 3.092 to 3.443)	> 0.99
**12 months**				
Mild vs. moderate	1.764 (− 0.587 to 4.115)	0.216	−0.094 (− 2.072 to 1.884)	> 0.99
Moderate vs. severe	0.837 (− 2.707 to 4.380)	> 0.99	1.857 (− 1.153 to 4.868)	0.416
Mild vs. severe	2.601 (− 1.175 to 6.376)	0.295	1.764 (− 1.443 to 4.971)	0.560
**24 months**				
Mild vs. moderate	1.768 (− 0.777 to 4.313)	0.286	0.157 (− 2.146 to 2.460)	> 0.99
Moderate vs. severe	−1.158 (− 4.913 to 2.597)	> 0.99	−0.235 (− 3.664 to 3.194)	> 0.99
Mild vs. severe	0.610 (− 3.415 to 4.635)	> 0.99	−0.078 (− 3.756 to 3.601)	> 0.99

Adjusted values were obtained by controlling for age, sex, body mass index, and preoperative OKS

All observed between-group differences were below the reported MCID threshold for OKS

*CTFS* coronal tibiofemoral subluxation, *OKS* Oxford Knee Score, *UKA* unicompartmental knee arthroplasty

### Preoperative radiographic characteristics

JLCA differed among the CTFS groups, with post-hoc analyses showing higher values in the severe group compared with both the mild and moderate groups (both *p* < 0.001: Table 4). LDFA showed significant group differences as well, with lower values in the moderate and severe groups compared with the mild group (*p* = 0.001 and *p* = 0.005, respectively: Table 4). CTFS severity showed a modest but significant correlation with JLCA (ρ = 0.29, *p* < 0.001).

**Table 4:** Preoperative radiographic characteristics

	Total	Mild CTFS group	Moderate CTFS group	Severe CTFS group	*p*-value (Kruskal–Wallis)	Post-hoc(Bonferroni)
FTA (°)	1.7 ± 3.2	1.4 ± 2.9	1.6 ± 3.2	2.6 ± 3.5	0.089	*N*.S.
LDFA (°)	81.8 ± 3.9	82.7 ± 2.1	81.5 ± 4.8	81.3 ± 2.4	< 0.001	mild vs. moderate: *p* = 0.001 mild vs. severe: *p* = 0.005
MPTA (°)	85.3 ± 4.1	85.7 ± 2.3	85.1 ± 5.0	85.5 ± 2.4	0.280	N.S.
JLCA (°)	5.1 ± 2.3	4.5 ± 2.0	5.2 ± 2.3	6.6 ± 2.4	< 0.001	mild vs. moderate: *p* = 0.007 mild vs. severe: *p* < 0.001 moderate vs. severe: *p* < 0.001
PTS (°)	8.4 ± 3.2	8.3 ± 3.3	8.4 ± 3.1	8.6 ± 3.4	0.835	N.S.

Data are presented as mean ± standard deviation (SD). p-values are based on the Kruskal–Wallis test. Post-hoc pairwise comparisons were performed using Bonferroni-adjusted tests

*CTFS* coronal tibiofemoral subluxation, *FTA* femorotibial angle, *LDFA* lateral distal femoral angle, *MPTA* medial proximal tibial angle, *JLCA* joint line convergence angle, *PTS* posterior tibial slope, *N.S.* not significant

### Bearing thickness

Mean bearing thicknesses did not differ significantly among the mild, moderate, and severe groups (3.6 ± 0.8, 3.7 ± 0.8, and 3.7 ± 0.9 mm, respectively; *p* = 0.428). No significant correlation was observed between CTFS severity and bearing thickness (ρ = 0.08, *p* = 0.083).

## Discussion

The important finding of this study is that postoperative short-term clinical outcomes were not affected by the presence or magnitude of CTFS. The severe CTFS group demonstrated numerically smaller early improvements; however, no significant differences were observed among the groups, and all groups achieved similar improvement at 2 years. The results suggest that patients with AMOA and correctable deformity of CTFS should not be considered a contraindication for mobile-bearing UKA.

Several studies have reported favorable long-term outcomes even in patients with severe varus deformity [12, 13], suggesting that deformity alone may not preclude successful UKA. However, the influence of CTFS remains less clearly understood. In the present cohort of patients with AMOA, CTFS severity was associated with JLCA and LDFA. JLCA and LDFA may both reflect morphological characteristics of the lateral femoral condyle, suggesting a potential relationship between subluxation and underlying bone morphology. However, differences in CTFS did not translate into variations in overall limb alignment. As only patients with correctable alignment were included, this may have influenced the findings. Although the correlation between CTFS severity and JLCA was statistically significant, the strength of the association was modest, suggesting limited clinical impact of this relationship alone.

Previous studies have suggested that pronounced CTFS may affect intraoperative soft tissue balance, including the need for thicker inserts [4]. In the present study, CTFS severity did not affect intraoperative balancing, as reflected by the absence of differences in the bearing thickness selected during surgery. This finding supports the concept that CTFS primarily reflects static joint morphology rather than true functional instability. Restoration of ligament balance in mobile-bearing UKA may help accommodate preoperative subluxation. Because mobile-bearing UKA allows a certain degree of self-alignment and congruent articulation during motion, preoperative coronal subluxation may have less influence on postoperative load distribution and bearing selection when deformity is functionally correctable. These findings suggest that in patients with AMOA, CTFS alone may not represent a contraindication to mobile-bearing UKA and should not preclude its use when appropriate indications are met. Prior studies reporting adverse outcomes often included knees with residual or uncorrectable subluxation [6, 14], potentially representing a distinct biomechanical scenario from the correctable deformities evaluated in our cohort. This difference may partly explain the discrepancy in clinical outcomes between studies.

Previous studies have reported that preoperative CTFS correlates with postoperative CTFS, although a certain degree of correction can be achieved during surgery [15]. However, in cases where CTFS does not correct with manual valgus stress preoperatively, substantial residual CTFS may persist after surgery and potentially contribute to postoperative pain [6]. It has also been reported that postoperative CTFS does not improve spontaneously over time [16]. Moreover, Kamenaga et al. demonstrated that residual CTFS after UKA may contribute to the progression of lateral compartment osteoarthritis [14], and Siriwanarangsun et al. similarly reported progression of cartilage degeneration in the lateral central region [17]. These findings raise concern regarding the potential long-term biomechanical consequences of residual subluxation. Together, these reports highlight the need for careful patient selection. In the present study, changes in subluxation under valgus stress were not evaluated; however, only patients whose alignment was manually correctable with valgus stress were included, and short-term outcomes were favorable. Nevertheless, because our results are limited to short-term follow-up, long-term clinical outcomes and radiographic progression warrant further investigation. Therefore, within the limitations of short-term follow-up, CTFS alone may not preclude candidacy for mobile-bearing UKA when standard indications are present, but should be interpreted in the context of ligament balance and the presence of subluxation on lateral radiographs when making surgical decisions.

The measurement of CTFS remains controversial because methods vary among studies and clear anatomical landmarks are still lacking [18–24]. Greif et al. demonstrated in their systematic review that no standardized technique exists for assessing coronal tibiofemoral subluxation, highlighting the need for a reproducible and consistent approach [2]. In this study, CTFS was measured according to previously described methods by assessing the displacement between the femoral and tibial articular surfaces while excluding osteophytes [1, 21]. Although alternative measurement approaches have been reported [20, 23, 24], these differences underscore the need for a consistent and reproducible method. To reduce the influence of individual knee size, we used a ratio-based approach normalized to tibial plateau width. However, consensus on the optimal measurement method has not been established, and further studies are warranted.

This study has several limitations. First, CTFS was assessed only preoperatively, and postoperative radiographic evaluations were not performed longitudinally. Residual postoperative CTFS was therefore not evaluated, although previous studies have suggested that persistent subluxation may influence clinical outcomes. The follow-up duration of two years may also be insufficient to fully assess long-term clinical outcomes. Alignment was not assessed using full-length lower limb radiographs, which limits the precision of detailed alignment analysis. Moreover, all procedures were performed by high-volume surgeons at a single center, which may introduce performance bias and limit extrapolation to other surgical settings. Because only mobile-bearing UKA was evaluated, the generalizability of these findings to fixed-bearing designs or other implant systems remains uncertain. The inclusion of only knees with correctable deformities may have introduced selection bias and limits the generalizability of our findings to more complex cases. The correctability of CTFS in the preoperative setting was not evaluated in this study. This distinction may be clinically important. In addition, bilateral cases were included, and both knees from the same patient were analyzed, which may have introduced non-independence of observations. However, as OKS was assessed independently for each knee, the impact of this issue is likely to be limited. Statistical methods accounting for within-patient correlation, such as mixed-effects modeling, may further strengthen future analyses. Longitudinal mixed-effects modeling may also provide a more robust approach for repeated postoperative OKS measurements over time. Missing PROM data and the use of available-case analysis may have introduced selection bias, although follow-up rates were acceptable across groups. Exclusion of knees requiring revision surgery or reoperation within the follow-up period may have introduced survivorship bias. Although measurement error cannot be entirely excluded given the limited interobserver validation sample, reliability was acceptable based on ICC. Given the retrospective nature of the study, unmeasured confounding and inherent selection bias cannot be fully excluded, and an a priori sample size calculation was not performed. The relatively small number of knees available at 2-year follow-up in the severe CTFS group may also have increased the risk of type II error. Despite these limitations, the relatively large cohort strengthens the robustness of the analyses. Future studies incorporating responder analyses based on MCID thresholds may further enhance clinical interpretability.

## Conclusion

Preoperative CTFS was not associated with worse outcomes in mobile-bearing UKA for AMOA with correctable deformity. Within the setting of correctable deformity and appropriate patient selection, preoperative CTFS was not associated with inferior 2-year outcomes. Longer-term survivorship and radiographic progression analyses are warranted.

## Supplementary Information

Below is the link to the electronic supplementary material.

Supplementary Material 1

## Data Availability

The datasets generated and/or analyzed during the current study are available from the corresponding author on reasonable request.
