# Supplementary material for: Preoperative coronal tibiofemoral subluxation does not affect 2-year outcomes after mobile-bearing medial unicompartmental knee arthroplasty for anteromedial osteoarthritis
Source: Arch Orthop Trauma Surg. 2026 Oct 29;146(1):387. doi: 10.1007/s00402-026-06530-x (PMC13719811; doi:10.1007/s00402-026-06530-x)
Supplement: Supplementary file 1 [file 402_2026_6530_MOESM1_ESM.docx]

Supplementary Table 1. Details of reoperation or revision cases and their CTFS distribution

|  | **Type of reoperation** | **reason** | **CTFS (%)** | **group** |
| --- | --- | --- | --- | --- |
| Case 1 | bearing exchange | bearing dislocation | 8.8 | moderate |
| Case 2 | bearing exchange | bearing dislocation | 4.7 | mild |
| Case 3 | bearing exchange | bearing dislocation | 8.7 | moderate |
| Case 4 | bearing exchange | bearing dislocation | 9.1 | moderate |
| Case 5 | debridement | periprosthetic joint infection | 8.3 | moderate |
| Case 6 | debridement | periprosthetic joint infection | 10.5 | severe |
| Case 7 | revision to TKA | periprosthetic fracture | 6.9 | moderate |
| Case 8 | revision to TKA | periprosthetic fracture | 12.3 | severe |
| Case 9 | revision to TKA | persistent pain | 6.9 | moderate |
| Case 10 | revision to TKA | persistent pain | 7.0 | moderate |

CTFS, coronal tibiofemoral subluxation; TKA, total knee arthroplasty.

Supplementary Table 2. Follow-up rates for Oxford Knee Score across CTFS groups

|  | Mild CTFS  group | Moderate CTFS group | Severe CTFS group |
| --- | --- | --- | --- |
| Preoperative | 114 / 134 (85.0%) | 228 / 265 (86.0%) | 45 / 58 (77.6%) |
| 3 months | 123 / 134 (91.8%) | 240 / 265 (90.6%) | 51 / 58 (87.9%) |
| 12 months | 122 / 134 (91.0%) | 229 / 265 (86.4%) | 48 / 58 (82.8%) |
| 24 months | 115 / 134 (85.8%) | 221 / 265 (83.4%) | 48 / 58 (82.8%) |

CTFS, coronal tibiofemoral subluxation.
